# New anatomical reference systems for the bones of the foot and ankle complex: definitions and exploitation on clinical conditions

**DOI:** 10.1186/s13047-021-00504-5

**Published:** 2021-12-20

**Authors:** Michele Conconi, Alessandro Pompili, Nicola Sancisi, Alberto Leardini, Stefano Durante, Claudio Belvedere

**Affiliations:** 1 Department of Industrial Engineering – DIN, University of Bologna, Viale del Risorgimento 2, 40136 Bologna, Italy; 2 Movement Analysis Laboratory, IRCCS Istituto Ortopedico Rizzoli, via di Barbiano 1/10, 40136 Bologna, Italy

**Keywords:** Anatomical reference system, Foot and ankle, Joint morphology

## Abstract

**Background:**

A complete definition of anatomical reference systems (ARS) for all bones of the foot and ankle complex is lacking. Using a morphological approach, we propose new ARS for these bones with the aim of being highly repeatable, consistent among individuals, clinically interpretable, and also suited for a sound kinematic description.

**Methods:**

Three specimens from healthy donors and three patients with flat feet were scanned in weight-bearing CT. The foot bones were segmented and ARS defined according to the proposed approach.

To assess repeatability, intra class coefficients (ICC) were computed both intra- and inter-operator.

Consistency was evaluated as the mean of the standard deviations of the ARS position and orientation, both within normal and flat feet.

Clinical interpretability was evaluated by providing a quantification of the curvature variation in the medial-longitudinal and transverse arches and computing the Djiann-Annonier angle for normal and flat feet from these new ARS axes.

To test the capability to also provide a sound description of the foot kinematics, the alignment between mean helical axes (MHA) and ARS axes was quantified.

**Results:**

ICC was 0.99 both inter- and intra-operator.

Rotational consistency was 4.7 ± 3.5 ° and 6.2 ± 4.4° for the normal and flat feet, respectively; translational consistency was 4.4 ± 4.0 mm and 5.4 ± 2.9 mm for the normal and flat feet, respectively. In both these cases, the consistency was better than what was achieved by using principal axes of inertia.

Curvature variation in the arches were well described and the measurements of the Djiann-Annoier angles from both normal and flat feet matched corresponding clinical observations.

The angle between tibio-talar MHA and ARS mediolateral axis in the talus was 12.3 ± 6.0, while the angle between talo-calcaneal MHA and ARS anteroposterior axis in the calcaneus was 17.2 ± 5.6, suggesting good capability to represent joint kinematics.

**Conclusions:**

The proposed ARS definitions are robust and provide a solid base for the 3-dimensional description of posture and motion of the foot and ankle complex from medical imaging.

**Supplementary Information:**

The online version contains supplementary material available at 10.1186/s13047-021-00504-5.

## Background

In human joints in general, and more specifically in the foot and ankle complex, a careful definition of relevant anatomical reference systems (ARS) is essential for the quantification of the absolute and relative position and orientation of the bones, thus allowing the description of the foot posture and motion in healthy and pathological conditions. Robust definitions of ARS must guarantee high inter- and intra-operator repeatability, consistency over follow-up assessments and among similar subjects, clinical relevance and interpretability, and a good alignment with joint axes of motion to optimize the kinematic description [1–3].

Early definitions of foot-ankle related ARS were provided for gait analysis via stereophotogrammetric systems and were based on external markers, meant to represent corresponding anatomical landmarks [4, 5]. However, due to the complexity in the identification by palpation of externally accessible landmarks for all the foot bones [6], ARS were defined mostly for multi-bone segments [7] [8]. Furthermore, it was impossible to define the ARS for the talus due to the direct inaccessibility of its landmarks [9, 10]. This marker-based approach provided insight in the general posture of the foot during functional tasks but did not allow its complete kinematic description [6]. Also, identification of anatomical landmarks by external palpation resulted in a low ARS repeatability [11].

The development and dissemination of new biomedical imaging techniques, such as Weight Bearing CT (WBCT) scanners based on the cone-beam technology, is providing easier access to a complete 3D representation of the foot and ankle bones, opening the way to new approaches in ARS definition [12]. In the literature, alternative approaches are based on landmarks [13, 14], principal inertial axes [15, 16], morphological fitting of geometrical features [17, 18] or a combination of the previous techniques [19, 20]. Anatomical landmarks can provide good clinical interpretation [21], but they are limited by the operator-dependent accuracy and repeatability [22]. Principal inertial axes can be determined automatically and thus do not depend on the operator [23]; however, the resulting ARS may be not directly associable with anatomical planes [21] nor optimal for kinematic description [1], and axis orientation is not uniquely defined [24]. Another possible approach consists in defining clinically interpretable ARS for one bone (typically the tibia) and assuming the same orientation of the ARS for all the foot bones in a given scan, for instance in ankle neutral configuration [25]. This is a simple and fast method, particularly suitable to describe the relative kinematics, but unable to provide a clinical description of the foot posture [26]. Finally, the morphological approach allows the identification of functionally relevant features that, despite being still sensitive to the operator [1], are more repeatable with respect to punctual landmarks thanks to the wider dimension of the fitting region. Furthermore, the axes identified through morphological fitting may provide a good approximation of the joint axes of motion, as shown for the tibiotalar and subtalar joints [17, 27]. Despite its potential, this latter approach is currently applied to the talus bone only and its application has not been fully exploited.

The variety and the number of the available approaches, also reflected in the number of different clinical measures to assess foot posture [28], suggest that a robust definition of ARS for all the bones of the foot and ankle is still lacking, making it difficult to perform a thorough comparison of different data from the literature.

The goal of the present study is to propose a new definition of ARS for the foot and ankle bones using a morphological approach mainly based on the shape of the articular surfaces. More specifically, we targeted ARS definitions that are: (a) highly repeatable; (b) consistent among individuals; (c) clinically interpretable; (d) optimized for the kinematic description. An algorithm is also provided that, given the appropriate geometrical features fitted on a 3D representation of the bones model, computes ARS for each bone in the foot and ankle complex.

## Methods

### Anatomical reference system (ARS) definitions

For each bone in the foot, the approach requires the operator to identify articular surfaces or other bone elements, to which simple geometrical features (plane, sphere, circle, cylinder) are fitted. To minimize the amount of geometrical feature to be manually identified and depending on the bone, ARS definition may also rely on principal axes of inertia and bone centroid, automatically identified from the corresponding 3D bone model. A complete list of all the features for each bone is reported in Table 1. ARS for bones not included in Table 1 rely uniquely on principal inertia axes. In this work, features identification and geometrical fitting were performed in Geomagic Studio 2012. Given the parameters describing the fitted features (Table 1) and a 3D model of the bones, ARS were computed automatically through a Matlab routine provided with this paper (Additional material: numerical routine for the ARS definition).

**Table 1:** List of geometrical features to be identified for each bone, together with the corresponding parameters required by the proposed numerical routine

Bones	Features	Parameters
Tibia	Cylinder fitted on the tibial plafond, i.e. the surface articulating with the talus excluding the medial malleolus.	Centre of medial base of the cylinder
		Centre of lateral base of the cylinder
	Circumference fitted on the most proximal cross-section available of the diaphysis	Centre of the circumference
Fibula	Plane fitted on the fibulo-talar articular surface	Normal to the plane
		Centroid of the selected articular surface
	Circumference fitted on the most distal cross-section of the diaphysis	Centre of the circumference
Talus	Cylinder fitted on the superior aspect of the trochlea tali, i.e. excluding the medial and lateral facies malleolaris	Centre of medial circumference
		Centre of lateral circumference
Calcaneus	Sphere fitted on both the anterior and middle talar facets	Centre of the sphere
	Sphere fitted on the posterior talar facet	Centre of the sphere
	Plane fitted on the surface articulating with the cuboid	Normal to the plane
Navicular	Sphere fitted on the articular surface between the navicular and talus	Centre of the sphere
Medial cuneiform	Plane fitted on the anterior surface articulating with the first metatarsal	Normal to the plane
	Plane fitted on the lateral surface articulating with intermediate cuneiform and second metatarsal	Normal to the plane
Intermediate cuneiform	Plane fitted on the anterior surface articulating with the second metatarsal	Normal to the plane
	Plane fitted on the posterior surface articulating with the navicular	Normal to the plane
	Plane fitted on the medial surface articulating with the medial cuneiform	Normal to the plane
	Plane fitted on the lateral surface articulating with the lateral cuneiform	Normal to the plane
Lateral cuneiform	Plane fitted on the anterior surface articulating with the third metatarsal	Normal to the plane
	Plane fitted on the posterior surface articulating with the navicular	Normal to the plane
	Plane fitted on the medial surface articulating with lateral cuneiform and second metatarsal	Normal to the plane
	Plane fitted on the lateral surface articulating with the cuboid	Normal to the plane
Cuboid	Plane fitted on the anterior surface articulating with the fourth and the fifth metatarsal	Normal to the plane
	Plane fitted on medial surface articulating with lateral cuneiform and navicular	Normal to the plane

Our approach relies on right-handed reference system for both right and left feet. As a general rule, the x axis points anteriorly, the y axis proximally and the z axis points to the right, thus laterally for right feet and medially for left feet.

For brevity, the ARS definition is presented here only for representative bones. The complete list of ARS definition is provided in the supplementary material (Additional material: full list of ARS definitions).

#### Tibia

**Fig. 1:**
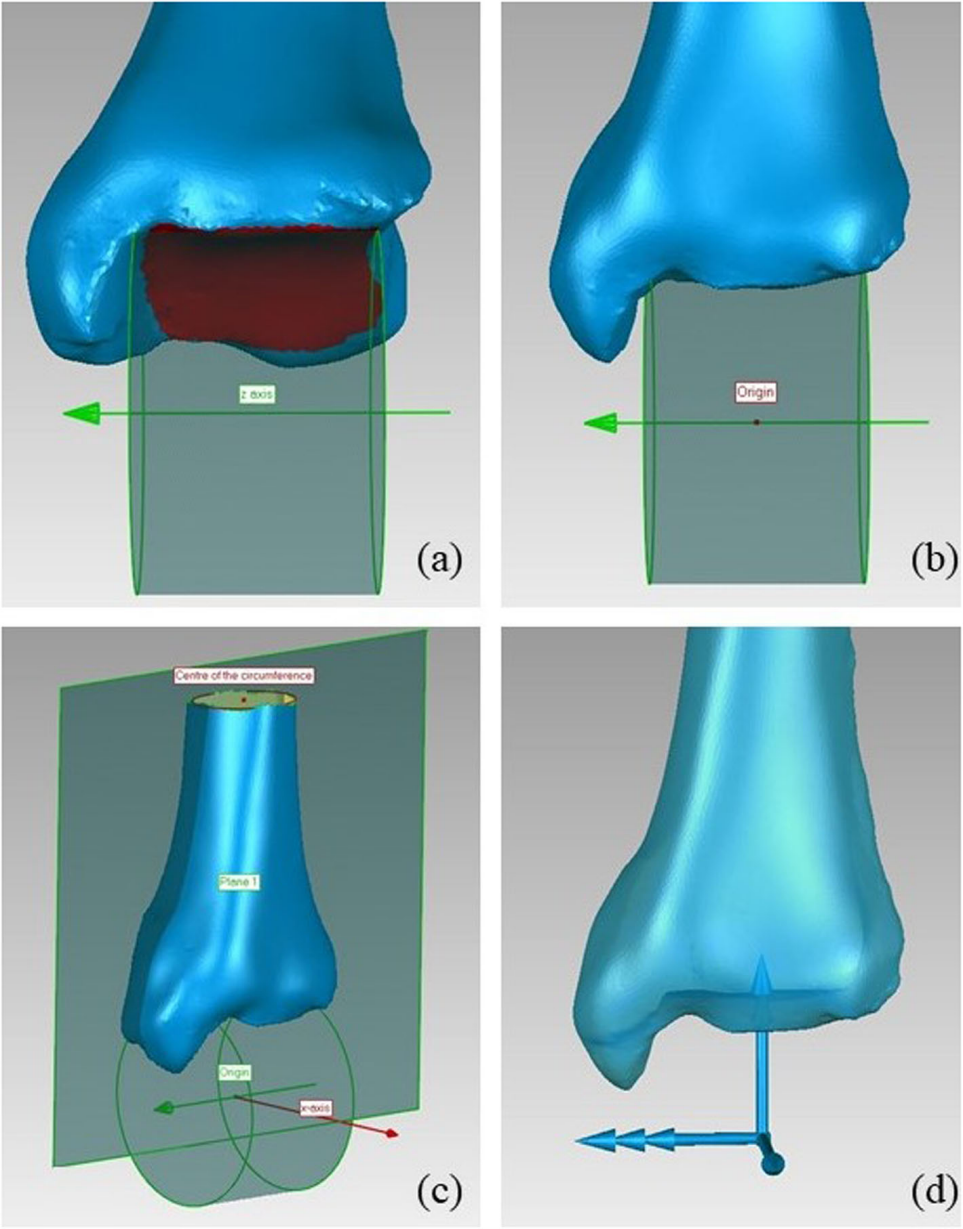
Definition of the tibia ARS: a) cylinder fitted on the tibial plafond and the z axis; b) origin; c) x axis definition; d) final ARS. In this and following figures a left foot is considered; the portion of articular surfaces used for the fitting of geometrical feature is represented in red; the construction curves are in green; the final x, y, and z axes of the ARS are in blue, represented by one, two and three arrows respectively

- The z axis is coincident with the axis of the cylinder fitted on the tibial plafond, pointing to the right;

- the origin is at the midpoint of the height of the same cylinder;

- the x axis is normal to the plane defined by z axis and the centre of the circumference fitted on the most proximal portion available of diaphysis, pointing anteriorly;

- the y axis is orthogonal to x and z axes, pointing proximally (Fig. 1).

#### Talus

**Fig. 2:**
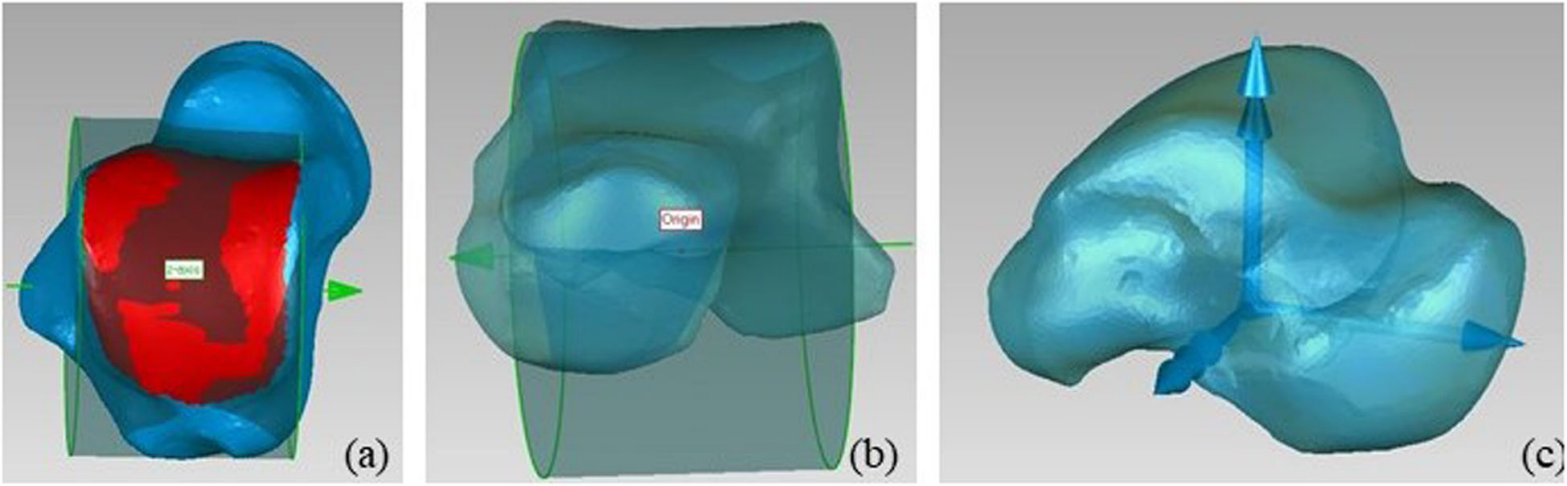
Definition of the talus ARS: a) cylinder fitted on trochlea tali; b) origin; c) final ARS

- The z axis is coincident with the axis of the cylinder fitted on the trochlea tali, pointing to the right;

- the origin is at the midpoint of the height of the same cylinder;

- as for the y axis, the talus principal axes of inertia are computed; the axis forming the smallest angle with the tibia x axis is chosen and its cross product with the z axis is taken, pointing proximally.

- the x axis is orthogonal to the y and z axes, pointing anteriorly (Fig. 2).

#### Calcaneus

**Fig. 3:**
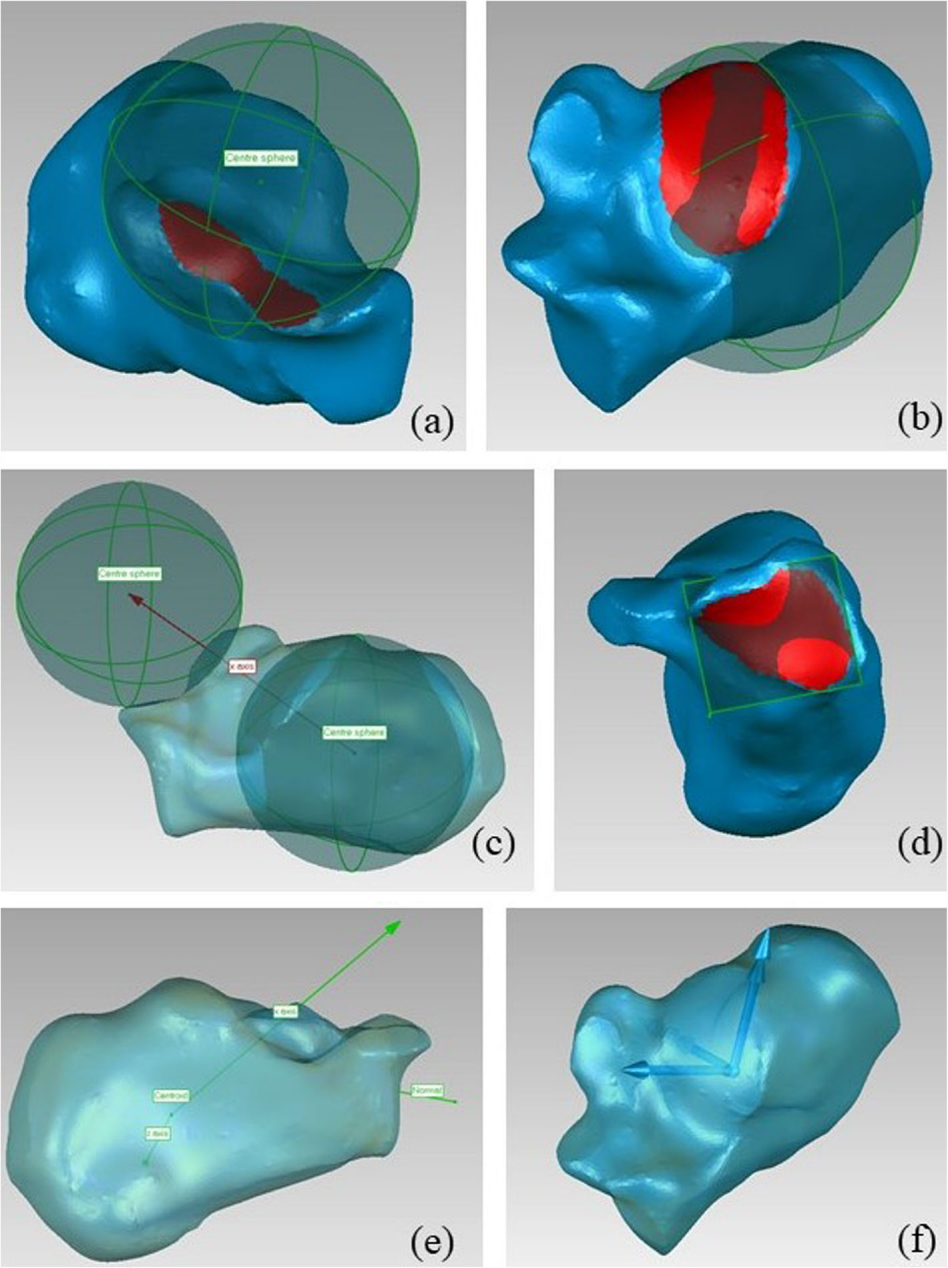
Definition of the calcaneus ARS: a) sphere fitted on anterior and middle talar facets; b) sphere fitted on posterior talar facet; c) x axis definition; d) plane fitted to the calcaneo-cuboidal articular surface; e) definition of the z axis; f) final ARS

- The origin is coincident with the bone centroid;

- the x axis is parallel to the line through the centers of the spheres fitted on articular surfaces (see Table 1), pointing anteriorly;

- the z axis is defined as the cross product between the x axis and the normal to the plane fitting to the articulating surface between calcaneus and cuboid, pointing to the right.

- the y axis is orthogonal to the x and z axes, pointing proximally (Fig. 3).

#### Navicular

**Fig. 4:**
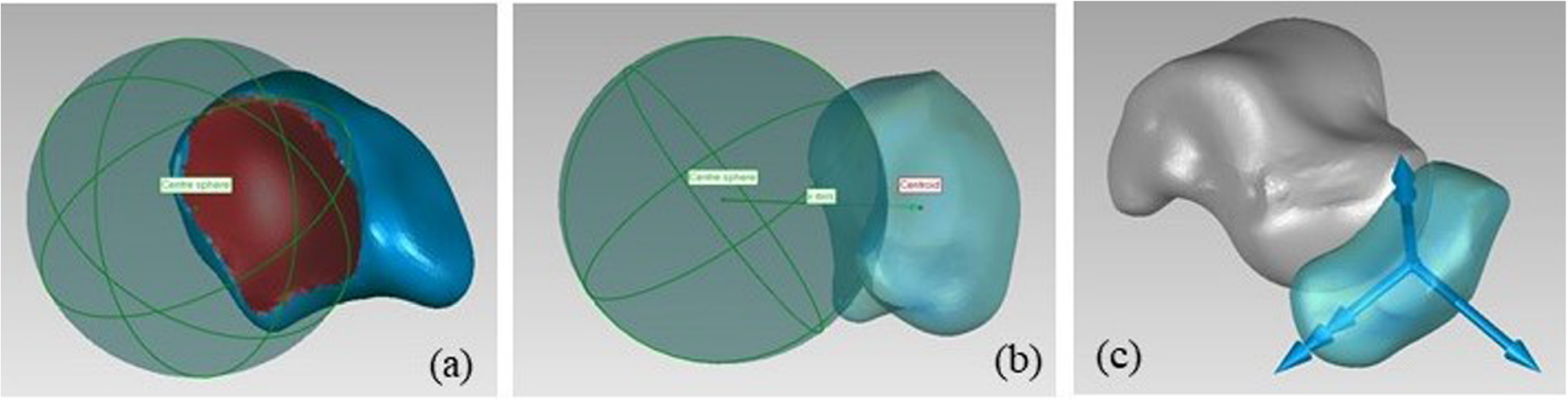
Definition of the navicular ARS: a) sphere fitted on the articular surface between navicular and talus; b) x axis definition; c) final ARS

- The origin is coincident with the bone centroid;

- the x axis is defined as the axis through the origin and the centre of the sphere fitted on articular surface between navicular and talus, pointing anteriorly;

- as for the y axis, the navicular principal axes of inertia are computed; the axis forming the smallest angle with the talus z axis is chosen and its cross product with the x axis is taken, pointing proximally;

- the z axis is orthogonal to the x and y axes, pointing to the right (Fig. 4).

#### Intermediate cuneiform

**Fig. 5:**
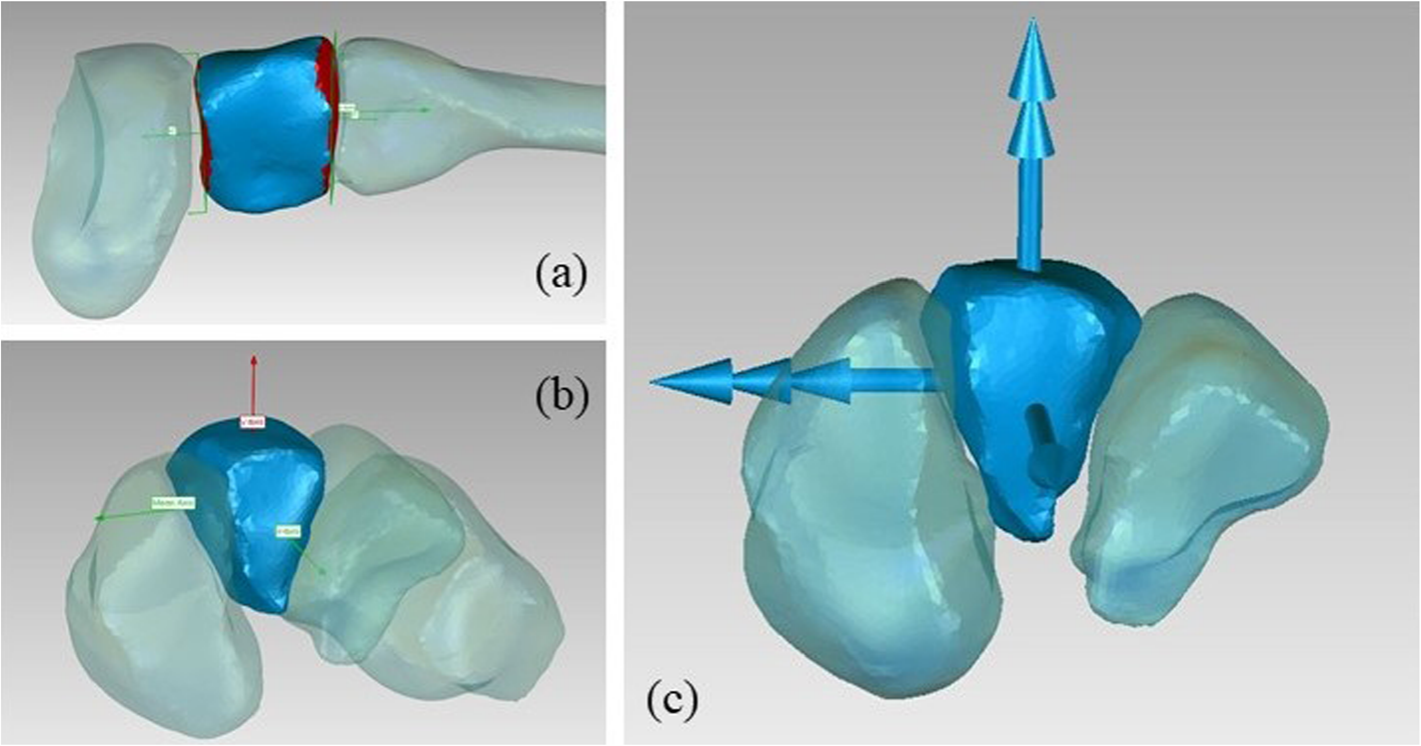
Definition of the intermediate cuneiform ARS: a) x axis definition; b) y axes definitions; c) final ARS

- The origin is coincident with the bone centroid;

- the x axis is the mean between the normal to the plane fitted on the anterior surface articulating with the second metatarsal and the normal to the plane fitted on posterior surface articulating with the navicular;

- as for the y axis, the mean between the normal to the plane fitted on the medial surface articulating with the medial cuneiform and the normal to the plane fitted on the lateral surface articulating with the lateral cuneiform is computed; its cross product with the x axis is taken, pointing proximally;

- the z axis is orthogonal to the x and y axes, pointing to the right.

- the y axis is orthogonal to x and z axes, pointing proximally (Fig. 5).

#### First metatarsal

**Fig. 6:**
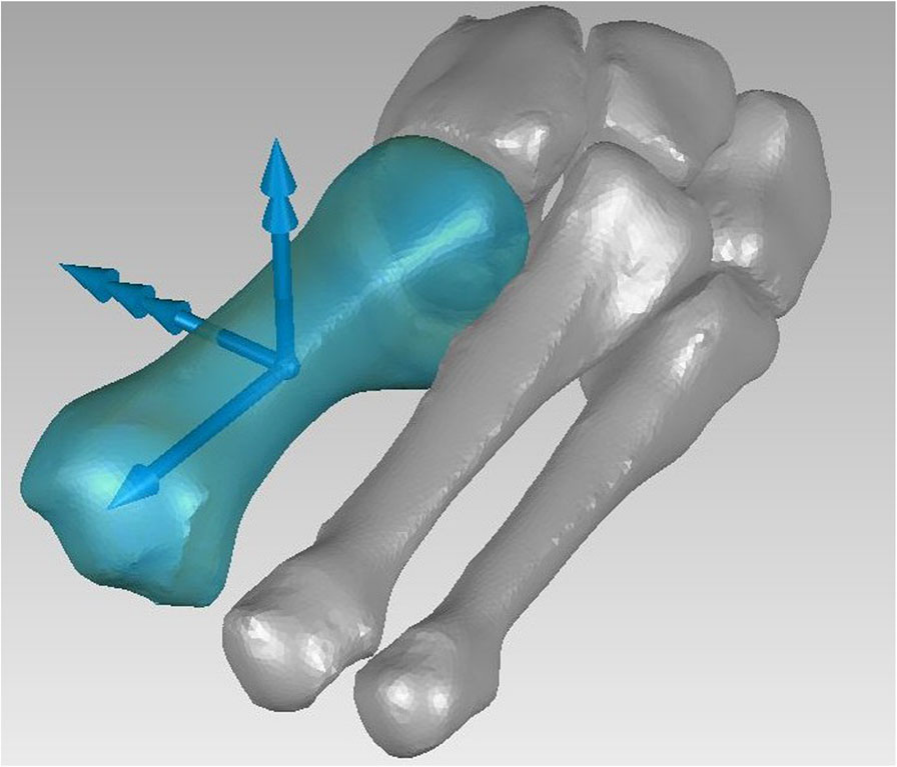
Final ARS for the first metatarsus

- The origin O is the centroid of the bone;

- the x axis is the bone first principal axis of inertia, pointing anteriorly, orientation is determined to minimize the angle with the medial cuneiform x axis;

- the y axis is the bone principal axis of inertia showing the minimum angle with the medial cuneiform y axis, pointing proximally;

- the z axis is orthogonal to the x and y axes, pointing to the right (Fig. 6).

### Data collection

We analysed two sets of feet: the first dataset consists of three fresh-frozen full lower limbs (male; age: 77.7 ± 7.8 years; height: 175.3 ± 5.8 cm; weight: 87.0 ± 15.7 Kg) from cadaver dissection with no history of previous pathologies and was meant to overcome limits due to ionizing radiation during CT scans (normal feet dataset). The second dataset includes three patients (female/male: 1/2; age: 47.3 ± 20.1 years; height: 175.7 ± 9.0 cm; weight: 74.7 ± 7.0 Kg) affected by flat feet taken from a previous study [29], to provide an initial exploitation on pathological feet of the presently proposed ARS (flat feet dataset).

**Fig. 7:**
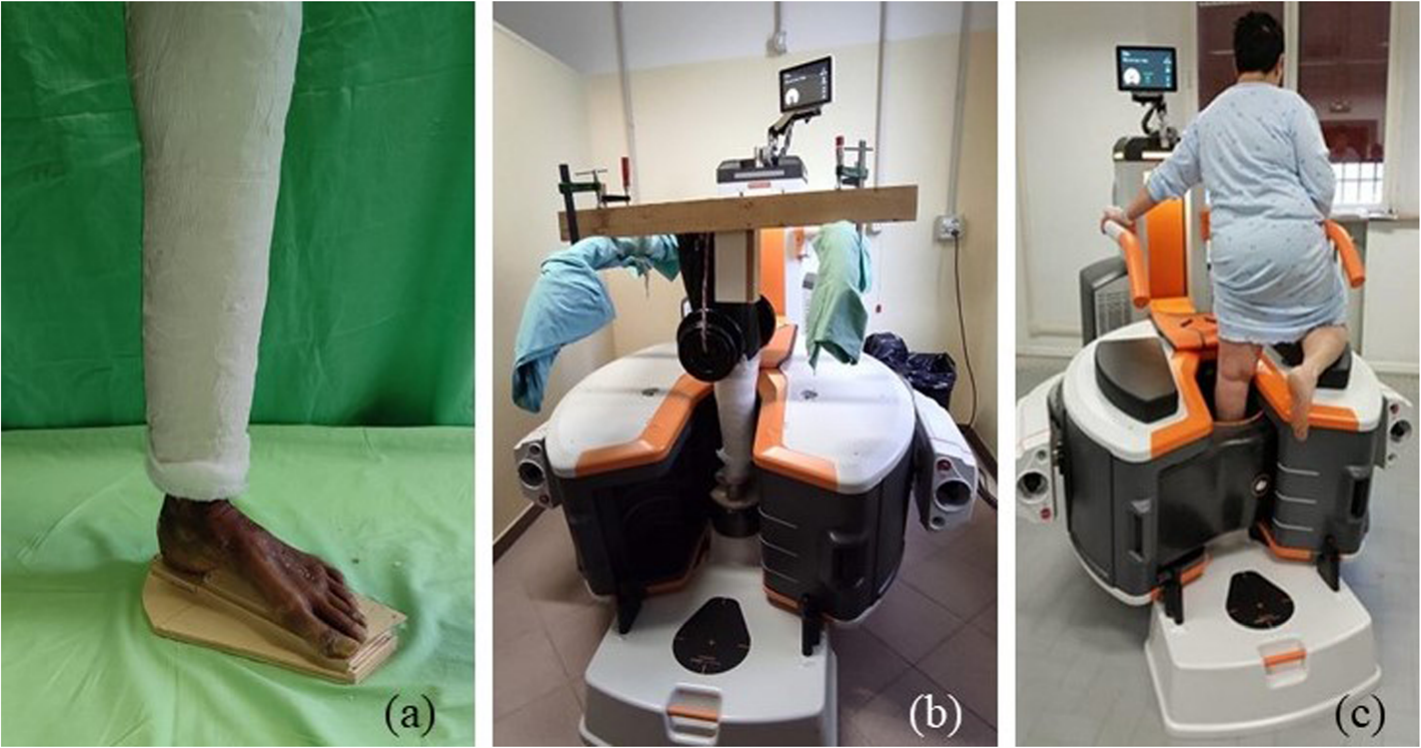
The experimental setup for the specimens, casted with the knee at full extension (a) and scanned in WBCT under load (b). Acquisition of foot scan for a typical patient with flat foot (c)

After a thawing period of 36 h, each specimen was casted with the knee in fully extended position (Fig. 7 a), leaving the foot and ankle free to move. Afterwards, each casted specimen was positioned within the acquisition bore of a WBCT scanner (Fig. 7 b) based on cone–beam technology (OnSight Extremity System, Carestream®, Rochester, NY-USA; 884-by-884-pixel resolution, 0.26 mm isotropic voxel size, 0.26 mm slice thickness and 230 × 230 mm field of view). Each leg was axially loaded by half the donor weight; this was achieved by few iron disks connected with a rope, hanging from the femur neck. Each leg was kept vertical by a wooden rig, attached to the CT frame, constraining the leg axis to remain vertical, without affecting the remaining degrees of freedom. The foot and ankle were scanned in this neutral configuration.

Patients were scanned with the same WBCT. The patient stood with one leg within the bore, with the foot and ankle in neutral configuration, while the contralateral leg was flexed and leaning on the outer surface of the scanner (Fig. 7 c).

**Fig. 8:**
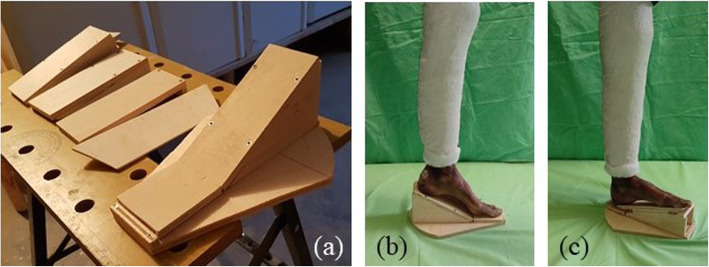
The wedges employed to impose foot posture during CT scanning (a) and their application in two cases: 30° plantar flexion (b) and a combination of 20° dorsi flexion and 10° supination (c)

To test the capability of the proposed ARS also to describe foot joint kinematics and as part of a wider study on the foot motion, 14 additional WBCT scans were acquired on the same day for each specimen, varying ankle dorsi-plantar flexion among five values (− 30°, − 10°, 0°,10°, and 20°) and foot prono-supination among three values (− 10°, 0°, and10°).. This was achieved through a set of wooden wedges (Fig. 8), while the leg was kept vertical and axial load maintained as previously described. A Dicom file was obtained for each scan by imposing a slice thickness of 0.26 mm, for a total of 960 CT images. From each of the 45 acquired scans, 3D models of all bones from the tibia to the metatarsals were obtained by a semi-automatic segmentation process (Medical Imaging Interaction Toolkit (MITK software 2003–2021, German Cancer Research Center - DKFZ).

### Evaluation of anatomical reference system robustness

To test the robustness of the ARS, their repeatability, consistency across subjects, clinical interpretability and optimization for the kinematic description were assessed.

#### Repeatability

Three different operators were enrolled and the ARS of the three specimens were defined by each of them. The operators were an undergraduate student, a PhD student, and a researcher, all with an education in biomechanics. The operators were all familiar with foot anatomy and with the employed software. All operators were instructed only using the information and definitions reported here and in the additional material. One operator repeated the ARS definition three times for each specimen. 2-way random effects intraclass correlations coefficients (ICC) were computed to assess both intra- and inter-operator repeatability [30], on the foot and ankle complex as a whole and for each bone. Translational and rotational variability among the ARS was also calculated, defined as the standard deviation among ARS positions and orientations as reconstructed inter- and intra-operator.

#### Consistency

The position and orientation of each bone with respect to the tibia ARS were computed in the neutral configuration of the foot and ankle, using a x-y-z cardanic sequence. For each bone and component, mean and standard deviation among the three subjects in each of the two datasets were computed. Consistency was evaluated as the mean of the standard deviations on the translational and rotational components separately. To cope with the lack of reference values for ARS consistency in the foot, reference systems totally based on principal inertial axes were also defined for each bone but the tibia and the fibula, whose bone model was limited by their different portions, associated to the different sizes of the subjects. The principal inertial axes of each bone were sorted and oriented to minimize the angle with the corresponding axis of the tibia ARS. Consistency analysis was repeated for inertial axis reference systems.

#### Clinical interpretability

The curvature of the medial longitudinal arch was described by the dorsi-plantar flexion angle (rotation about z-axis) of calcaneus, talus, navicular, medial cuneiform, and first metatarsus. The curvature of the transverse arch was described by the pronosupination angle (rotation about x-axis) of medial, intermediate, lateral cuneiform and cuboid. To compare our results with the clinical literature, the Djian-Annonier angle [28] was also calculated as the spatial angle between the x axes of the calcaneus and of the first metatarsal and compared with average data from the literature [31].

#### Optimization for the kinematic description

To test the capability of the proposed ARS to describe the foot kinematics, we focused on three articulations: the tibio-talar and talo-calcaneal joints, approximately rotating around a single axis and therefore often represented as hinge joints, and the talo-navicular joint, which can be approximated as a spherical pair. If our morphological approach correctly captures the main kinematic characteristics of each articulation, we should be able to establish a relation also between the ARS and the mean helical axis (MHA) as well as the mean pivot point (MPP) of joint motion, the former representing the single axis of rotation for hinge-like joints and the latter the centre of rotation of spherical-like joints that optimally describe the real three-dimensional motion of the considered articulations. This would provide an optimal decoupling of motion components, simplifying the clinical interpretation of the joint motion.

Joint motion was reconstructed from the 15 scans acquired with different dorsi-plantar flexion and pronosupination angles. The rototranslational transformations from the neutral to the other 14 ft postures were computed with an automatic ICP registration algorithm for each single bone. Eight foot motions were then simulated from these scans: three ankle dorsi-plantar flexions, differing by the pronosupination angle, and five foot pronosupination, differing by ankle dorsi-plantar flexion. The MHA and MPP were calculated from these motions according to Woltring [32]. Tibio-talar MHA was compared with the axis of the cylinder fitted on the talar trochlea, namely the z axis of the talar ARS, computing the angle between them. Talo-calcaneal MHA was compared with the axis through the centres of the two spheres fitting the anterior and posterior talo-calcaneal articulating surfaces, namely the x axis of the calcaneal ARS, computing the angle between them. Finally, the MPP of the talo-navicular motion was compared with the centre of the sphere fitted on the navicular surface articulating with the talus, lying on the x axis of the navicular ARS, computing the distance between them.

## Results

**Fig. 9:**
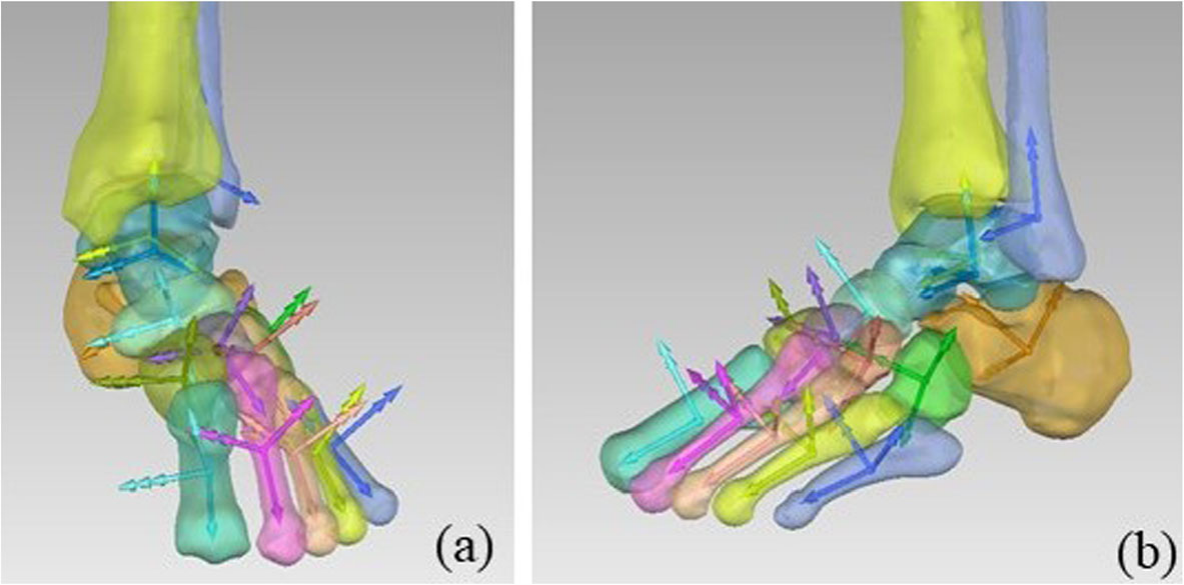
Overall representation of foot and ankle complex ARS for a representative specimen, in ankle neutral posture

**Fig. 10:**
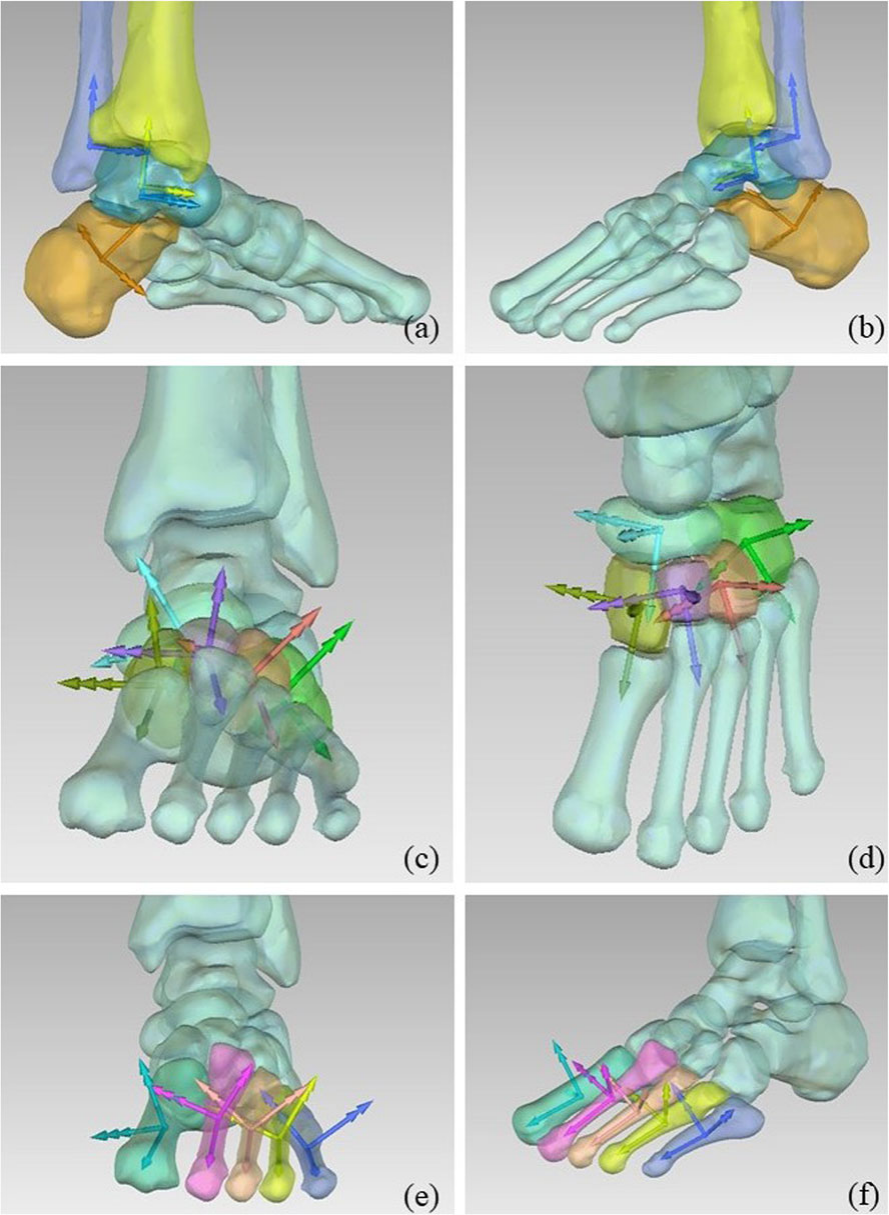
Representation of the ARS for the hindfoot bones (a and b), for the midfoot bones (c and d), and for the forefoot bones (e and f) for a representative specimen, in ankle neutral posture

The overall foot and ankle ARS are depicted in Fig. 9. To help their observation, ARS are also shown for the different groups of bones composing the foot and ankle complex: the hindfoot, the midfoot, and the forefoot (Fig. 10).

### Repeatability

The ICC values for intra-operator and inter-operator repeatability analyses were both 0.99 for the whole foot and ankle complex. The same values were obtained for the ARS of the single bones. The largest translation variability was 3.5 mm, while the largest rotational variability was 4.2°, both observed in the intra-operator analysis, for the fibula.

### Consistency

Consistency in orientation was 4.7 ± 3.5° while in position was 4.4 ± 4.0 mm for the normal feet dataset. For the flat feet dataset, consistency in orientation was 6.2 ± 4.4° while in position was 5.4 ± 2.9 mm.

In comparison, when considering the alternative anatomical reference systems based completely on the principal axes of inertia, consistency in orientation was 12.0 ± 12.0° while in position was 4.6 ± 4.1 mm for the normal feet dataset. For the flat feet dataset, consistency in orientation was 8.7 ± 8.0° while in position was 5.7 ± 3.6 mm.

A complete description of the average neutral posture of the foot and ankle complex for both the normal and flat feet datasets is reported in the additional material.

### Clinical interpretability

**Fig. 11:**
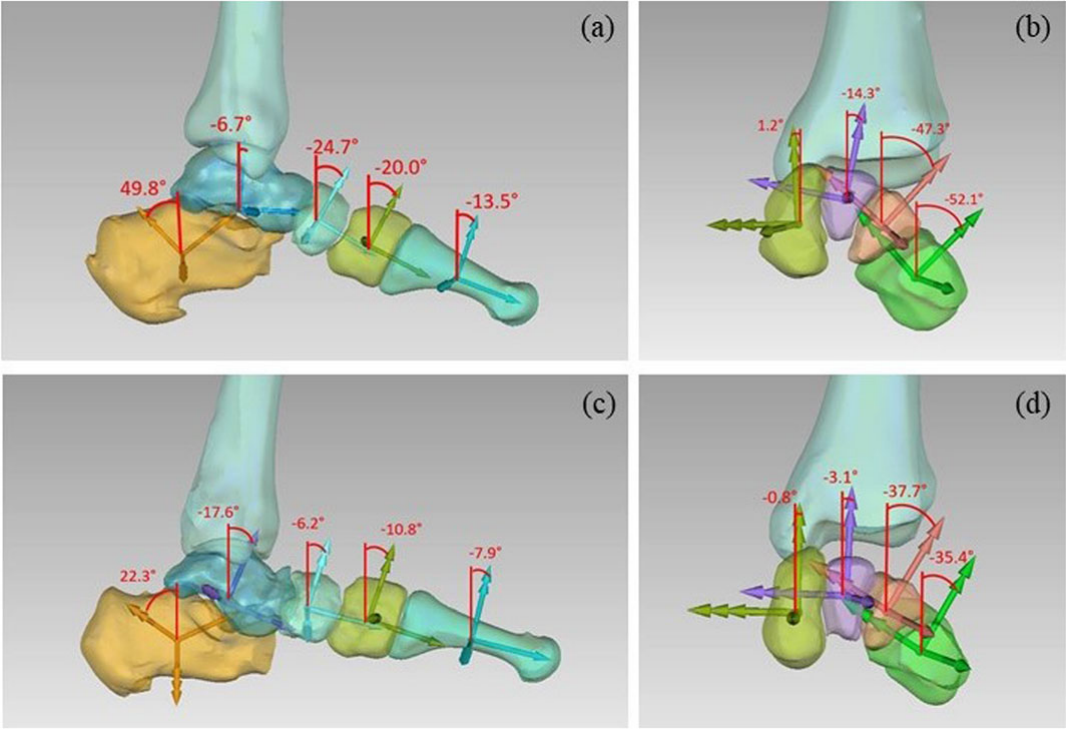
Quantification of longitudinal arch curvature in the sagittal plane for a specimen with no pathology (a) and a subject with flat feet (c); quantification of transverse arch curvature through pronosupination angle for a specimen with no pathology (b) and a subject with flat feet (d). The angular values are represented as projected on the plane of view for sake of convenience, however these are spatial angles obtained according to the described parametrization of bone orientation

The variation in the ARS orientation along the medial longitudinal arch and the transverse arch are depicted in Fig. 11 for one specimen and one flat foot. The numerical comparison between the two datasets for the two arches are reported in Tables 2 and 3. The collapse of the medial longitudinal arch is well represented by the dorsi-plantar flexion of the talo-calcaneal group, while the first ray dorsiflexed rotating about the talar head. The transverse arch also flattened and lowered.

**Table 2:** Quantification of 1 the medial longitudinal arch curvature

	Calcaneus	Talus	Navicular	Medial cuneiform	First metatarsal
Specimens without pathologies	51.2° ± 2.4	-6.3° ± 4.7	-24.4° ± 0.4	-17.4° ± 3.0	-12.2° ± 4.7
Subjects with flatfeet	30.6° ± 8.5	-14.5° ± 6.6	-19.4° ± 11.3	-9.1° ± 3.5	-3.1° ± 4.0

**Table 3:** Quantification of the transverse arch curvature

	Medial cuneiform	Intermediate cuneiform	Lateral cuneiform	Cuboid
Specimens without pathologies	3.9° ± 5.5	-9.0° ± 3.9	40.7° ± 4.8	-44.8° ± 6.5
Subject with flatfeet	5.2° ± 5.9	-1.1° ± 5.3	-36.8° ± 8.8	-32.8° ± 8.6

The estimated Djian-Annonier angle using the proposed ARS is depicted in Table 4, together with reference values for both normal and flat feet.

**Table 4:** The calculated Djian-Annonier angle versus reference values from the literature [31]

	Djian-Annonier angle	
	Spatial Estimated from ARS	Reference clinical values
Specimens without pathologies	122.1 ° ± 4.1	119° - 128°
Subject with flatfeet	134.7 ° ± 6.7	> 128°

### Optimization for the kinematic description

**Fig. 12:**
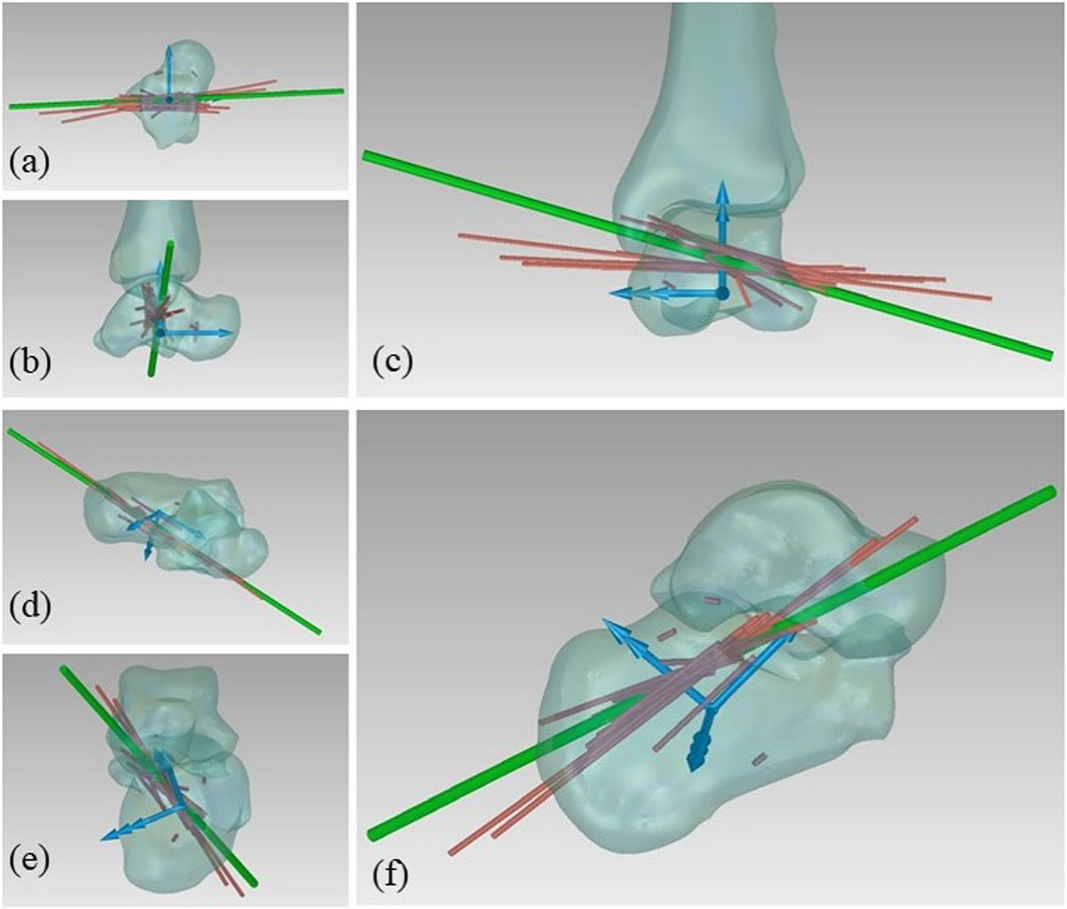
a) MHA of the tibio-talar motion in the talus ARS; b) MHA of the talo-calcaneal joint motion in the calcaneus ARS

The angle between the MHA and the z axis of the talar ARS was 12.3 ± 6.0. The angle between the MHA and the x axis of the calcaneal ARS was 17.2 ± 5.6. The orientation of the MHA with respect to the talar and calcaneal ARS are depicted in Fig. 12.

The mean distance between the centre of the sphere fitted on articular surface between navicular and talus and the MPP of the talo-navicular joint motion was 2.7 ± 0.4 mm.

## Discussion

The aim of this work was to define robust anatomical reference systems for each bone of the human foot and ankle complex. To this end, a procedure based mainly on morphological fitting of simple geometrical features on the articular surfaces is presented, also implemented in a complementary software code provided with the present paper.

Our analysis shows that the proposed definition is highly repeatable for all these bones, both inter- and intra- operator. Moreover, the identification of ARS is highly consistent among individuals, providing a solid representation of the three-dimensional posture of the foot and ankle complex. This representation is also clinically relevant, as shown for instance by the quantification of the curvature of the foot arches in normal and flat feet, whose values compared well with those observed in standard clinical measurements from planar radiographs. With respect to these, the present definitions clearly allow geometrical representations of position and orientation in three dimensions, thus extending standard measurements. Finally, the presented ARS are optimized for joint motion description, as proven by the alignment between mean helical axes and specific reference axes, particularly in the talar and calcaneal reference system. This suggests that the proposed ARS may also describe motion of the foot joints well, minimizing the apparent coupling among different motion components.

Although the proposed ARS may provide valuable clinical information, they are not meant to substitute standard radiographic inspection, which due to the simplicity in the setup, the diffusion and the established use will remain the gold standard for traditional clinical assessment of foot pathologies. Rather, the proposed ARS are intended to maximize the opportunities presented by the emerging techniques such as the WBCT, which makes it possible to investigate overall foot and ankle posture in three dimensions, opening the way to new 3D measures [33] and providing a solid framework for the investigation of foot motion.

While similar approaches have been previously proposed, particularly for the tibia and talus [12], a systematic use of morphological fitting to define ARS in the foot has not been previously reported in the literature. In comparison with other possible approaches, morphological fitting shows several advantages. With respect to virtual identification of anatomical landmarks, the operator must identify broader surfaces. Thus, the uncertainties introduced by the operator are smoothed by the dimension of the selected areas and by the fitting process. As a result, the approach presented is more repeatable and consistent than others based on virtual palpation of anatomical landmarks [21]. Also, the present ARS axes are optimally oriented with respect to axes of joint motion [1].

When compared with the fully automatic methods based only on principal axes of inertia [29, 33] for the definition of reference systems, the approach here presented has the advantage to be more clinically relevant in genreal [21], still showing a very high repeatability. In terms of consistency however, the presented ARS perform better, since the principal axes of inertia are indeed quite sensitive to bone geometry for axes different from the main one, particularly in case of long-cylindrical objects (such as the metatarsal bones), and in general in case of stocky objects such as cuneiforms or cuboid.

The proposed approach has limitations. Its application requires a full three-dimensional model for each bone of the foot and ankle complex, with the exception of the tibia and the fibula for which the distal portions are sufficient. The number of subjects analyzed needs to be increased to further test the consistency of the results. The manual identification of the necessary geometrical features and the calculation of all the ARS require some time. For this reason, we provide the code for the automatic computation of the ARS in the supplementary material, once the parameters of the fitted geometrical features have been defined by an operator. With this, the definition of the ARS for the whole foot and ankle complex takes roughly 30 min. Also, despite the excellent repeatability, the approach still depends to a certain extent on the operator. For both these reasons, future activities will aim at the full automatization of the process.

Finally, the present work represents the preliminary step toward an ongoing investigation of the foot kinematics under loads. Future activities will exploit the proposed ARS to describe the variation in the foot posture with ankle dorsi-plantar flexion and foot pronosupination thoroughly.

## Conclusion

According to a morphological approach, careful definitions of anatomical reference frames for all bones in the foot and ankle complex are proposed and exploited in a number of feet. These definitions proved to be robust, being found highly repeatable and consistent among individuals within the same population. Also, the proposed ARS are clinically interpretable, thus providing quantitative measures to be used in clinical studies. Finally, these definitions were shown to be suited for the description of joint kinematics. Therefore, the present approach provides a solid base for the 3-dimensional description of posture and motion of the foot and ankle skeletal structures.

## Supplementary Information

**Additional file 1.** Additional Material.

## Data Availability

Please contact author for data requests.

## Abbreviations

ARS: Anatomical reference system
ICC: Intra class coefficent
MHA: Mean helical axis
WBCT: Weight bearing CT
MPP: Mean pivot point
