# Supplementary material for: New anatomical reference systems for the bones of the foot and ankle complex: definitions and exploitation on clinical conditions
Source: J Foot Ankle Res. 2021 Dec 20;14:66. doi: 10.1186/s13047-021-00504-5 (PMC8686310; doi:10.1186/s13047-021-00504-5)
Supplement: Supplementary file 1 — Additional file 1. Additional Material. [file 13047_2021_504_MOESM1_ESM.docx]

New Anatomical Reference Systems for the Bones in the Foot and Ankle Complex: Definitions and Exploitation On Clinical Conditions

Additional Material

Table S1: Mean posture (orientation and position of each bone) for the healthy foot and ankle complex in neutral position

| Bone | Pronation-supination (x rotation) | Abduction-adduction (y rotation) | Dorsi-plantar flexion  (z rotation) | Antero-posterior  translation (x) | Distal-proximal translation (y) | Medio-lateral translation (z) |
| --- | --- | --- | --- | --- | --- | --- |
| Fibula | -3.0 ± 2.1 | 5.0 ± 2.3 | 4.5 ± 0.4 | -5.9 ± 1.4 | 9.0 ± 7.0 | -23.7 ± 1.2 |
| Talus | 2,1 ± 1.3 | 3.5 ± 3.3 | -6.4 ± 4.7 | 1.9 ± 0.6 | -9.0 ± 2.8 | 7.7 ± 0.9 |
| Calcaneus | 8.3 ± 6.2 | -22.2 ± 2.7 | 51.8 ± 2.4 | -14.2 ± 2.6 | -32.3 ± 0.2 | -10.2 ± 0.7 |
| Navicular | 31.8 ± 2.1 | -7.2 ± 7.8 | -24.4 ± 0.4 | 38.8 ± 0.3 | -11.7 ± 4.0 | 12.2 ± 4.1 |
| Medial Cuneiform | 3.9 ± 5.6 | -16.2 ± 2.7 | -17.4 ± 3.0 | 62.3 ± 0.7 | -19.6 ± 3.0 | 18.4 ± 6.1 |
| Intermediate Cuneiform | -9.0 ± 3.9 | 9.1 ± 3.9 | -15.5 ± 3.6 | 59.0 ± 0.3 | -10.9 ± 3.1 | 4.8 ± 5.0 |
| Lateral Cuneiform | -40.7 ± 4.8 | 20.5 ± 7.9 | -28.4 ± 1.8 | 54.6 ± 1.9 | -17.4 ± 1.7 | -7.5 ± 5.0 |
| Cuboid | -44.8 ± 6.5 | 33.0 ± 10.5 | -28.0 ± 2.1 | 38.4 ± 2.6 | -27.7 ± 2.2 | -16.5 ± 4.3 |
| Metatarsal 1 | 20.6 ± 6.9 | -22.5 ± 3.3 | -12.2 ± 4.7 | 102.1 ± 1.3 | -26.9 ± 1.1 | 29.5 ± 7.4 |
| Metatarsal 2 | -12.3 ± 3.4 | -10.3 ± 4.5 | -20.3 ± 1.0 | 100.3 ± 2.2 | -23.8 ± 1.0 | 6.7 ± 8.9 |
| Metatarsal 3 | -29.9 ± 5.4 | -8.4 ± 6.7 | -15.4 ± 1.7 | 95.7 ± 3.6 | -28.7 ± 0.5 | -6.8 ± 9.1 |
| Metatarsal 4 | -16.4 ± 2.3 | -5.0 ± 7.5 | -10.0 ± 2.3 | 88.2 ± 5.7 | -33.9 ± 1.6 | -19.3 ± 9.3 |
| Metatarsal 5 | -58.6 ± 13.0 | 1.3 ± 7.5 | -2.5 ± 2.2 | 74.6 ± 6.2 | -41.1 ± 2.5 | -33.0 ± 8.3 |

Table S2: Mean posture (orientation and position of each bone) for the flat foot and ankle complex in neutral position

| Bone | Pronation-supination (x rotation) | Abduction-adduction (y rotation) | Dorsi-plantar flexion  (z rotation) | Antero-posterior  translation (x) | Distal-proximal translation (y) | Medio-lateral translation (z) |
| --- | --- | --- | --- | --- | --- | --- |
| Fibula | 0.9 ± 1.1 | 6.7 ± 4.1 | 5.3 ± 2.2 | -6.2 ± 1.6 | 11.5 ± 5.0 | -22.8 ± 3.1 |
| Talus | 3.3 ± 0.7 | -5.3 ± 2.1 | -14.5 ± 6.6 | 0.5 ± 0.7 | -1.4 ± 1.3 | -1.4 ± 0.4 |
| Calcaneus | 45.6 ± 23.7 | -24.7 ± 2.0 | 30.6 ± 8.5 | -16.3 ± 3.6 | -19.7 ± 4.3 | -12.9 ± 3.4 |
| Navicular | 37.5 ± 3.2 | 12.6 ± 12-1 | -19.4 ± 11.3 | 33.4 ± 0.8 | -5.2 ± 2.7 | 5.1 ± 3.8 |
| Medial Cuneiform | 5.2 ± 5.9 | 11.0 ± 6.9 | -9.0 ± 3.5 | 56.5 ± 0.9 | -7.4 ± 4.6 | 0.6 ± 9.4 |
| Intermediate Cuneiform | -1.1 ± 5.3 | 32.9 ± 8.9 | -2.6 ± 8.1 | 49.2 ± 3.7 | 0.6 ± 6.7 | -8.8 ± 6.4 |
| Lateral Cuneiform | -36.8 ± 8.8 | 42.3 ± 8.2 | -16.2 ± 6.5 | 42.9 ± 4.0 | -4.6 ± 5.2 | -18.4 ± 5.3 |
| Cuboid | -32.8 ± 8.6 | 45.6 ± 8.3 | -14.8 ± 5.2 | 30.7 ± 2.9 | -15.9 ± 4.7 | -25.3 ± 5.6 |
| Metatarsal 1 | 18.8 ± 15.2 | 6.0 ± 4.8 | -3.2 ± 4.1 | 96.2 ± 1.9 | -5.2 ± 5.0 | -6.7 ± 11.2 |
| Metatarsal 2 | -15.1 ± 10.9 | 17.5 ± 5.1 | -9.6 ± 2.8 | 86.1 ± 5.1 | -3.6 ± 6.9 | -24.3 ± 10.4 |
| Metatarsal 3 | -25.3 ± 7.0 | 19.7 ± 4.7 | -4.8 ± 2.7 | 79.0 ± 6.1 | -7.8 ± 7.7 | -34.8 ± 9.7 |
| Metatarsal 4 | -10.6 ± 7.1 | 22.0 ± 4.2 | 1.6 ± 3.0 | 71.4 ± 5.9 | -11.7 ± 8.1 | -44.4 ± 9.5 |
| Metatarsal 5 | -38.2 ± 2.7 | 24.7 ± 5.0 | 8.5 ± 1.3 | 57.3 ± 7.4 | -17.7 ± 8.1 | -53.7 ± 8.6 |

Detailed Description Of Anatomical Reference System (ARS) Definition

In what follows a complete definition of the ARS of all the bone is presented. For each bone:

- Firstly, the geometrical features to be identified and the corresponding parameters for the numerical code are listed.
- Comments and suggestions for the correct selection of the surfaces for the fitting are then provided, when needed.
- Finally, the precise definition of the ARS construction is given

In general, all the feature proposed are quite stable, however it can be useful to test the effect of different selection, at least in an early stage. For the new user, we suggest identifying each required surfaces and the corresponding feature several times (at least 5) to get an estimation of the sensitivity of the method.

The Matlab routine here presented require that all parameters of the fitting features and all the bone STL files are provided at the same time. In other words, the routine is not designed to define single bone ARS. The routine is here presented in three version: one to define ARS for the whole foot, one for hindfoot only and one for hindfoot plus midfoot. Also, it is possible to chose whether define or not the ARS for the fibula.

For the sake of simplicity, principal axes of inertia and bone centroids are computed in the routine from the clouds of points corresponding to the vertices of the mesh of each bone. If the mesh is sufficiently fine and uniform, results are representative of an exact volumetric calculation. For this reason, we recommend avoiding coarse meshes.

Tibia

| **Features to be identified** | **Parameters for the input file for the numerical code** |
| --- | --- |
| Cylinder fitted on the tibial plafond, i.e. the surface articulating with the talus excluding the medial malleolus. | Centre of the medial base of the cylinder |
|  | Centre of the lateral base of the cylinder |
| Circumference fitted on the most proximal cross-section available of the diaphysis | Centre of the circumference |

The tibia plafond is the articular surface with the talus excluding the medial malleolus (fig. S1a – fig. S1b). Anterior and posterior selection should stop before the shoulder of the subchondral region, where the curvature changes in direction.

While the orientation of the fitted cylinder is very stable, the location of the origin can be quite sensitive to the tibia plafond selection, mainly in proximo-distal direction. For this reason, it is recommended to be consistent in the selection, taking particular care to the border of the selection.

The identification of the circumference fitted on the diaphysis is quite stable and the ARS definition is little sensitive to variation of this parameter.

*ARS definition*

- the z axis is coincident with the axis of the cylinder fitted on the tibial plafond, pointing to the right (fig. S1.c);

- the origin is at the midpoint of the height of the same cylinder (fig. S1.d);

- the x axis is normal to the plane defined by z axis and the centre of the circumference fitted on the most proximal portion available of diaphysis, pointing anteriorly (fig S1. e);

- the y axis is orthogonal to x and z axes, pointing proximally (fig. S1.f).


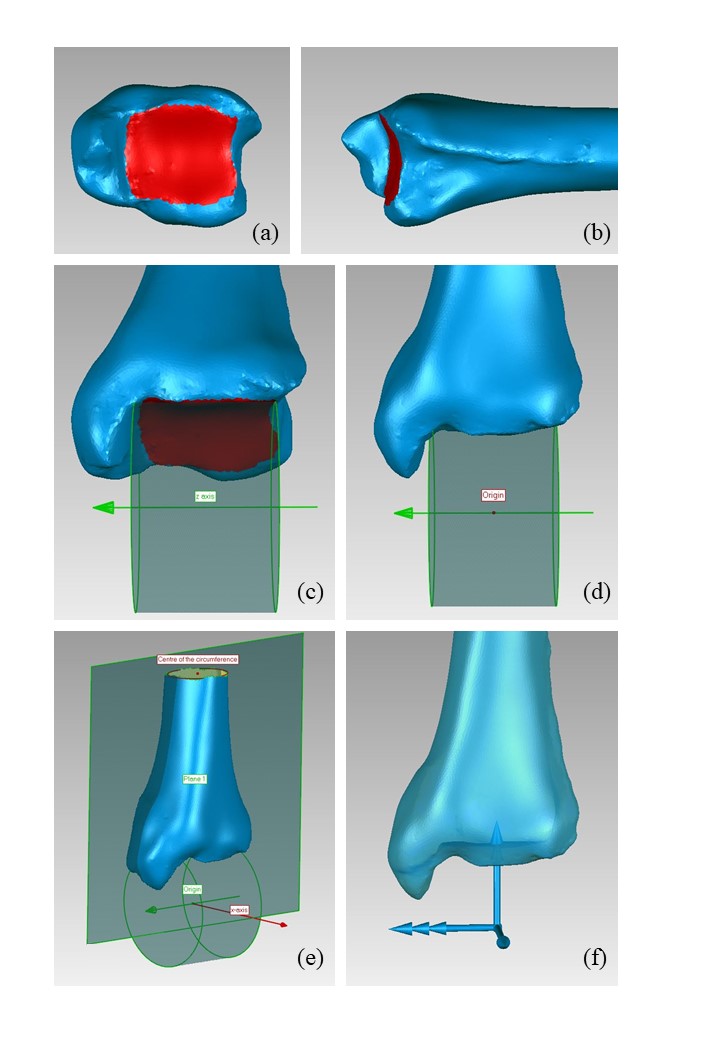


Figure S1: Definition of the tibia ARS: a) selection of the tibia plafond in axial view b) selection of the tibia plafond in lateral view c) cylinder fitted on the tibial plafond d) origin identification e) x-axis definition f) final ARS. In this and following figures a left foot is considered; the portion of articular surfaces used for the fitting of geometrical feature is represented in red; the construction curves are in green; the final x, y, and z axes of the ARS are in blue, represented by one, two and three arrows respectively.

Fibula

| **Features to be identified** | **Parameters for the input file for the numerical code** |
| --- | --- |
| Plane fitted on the fibulo-talar articular surface | Normal to the plane |
|  | Centroid of the selected area |
| Circumference fitted on the most distal cross-section of the diaphysis | Centre of the circumference |
| Circumference fitted on the most proximal cross-section available of the diaphysis | Centre of the circumference |

Fitting a plane on the fibula-talar articular surface (fig. S2. A-c) may seem a stretch. However, the plane is here used only to define the origin and orientation of the z axis and thus the impact of the quality of its fitting is minor on the ARS definition.

The identification of the circumferences fitted on the diaphysis is quite stable and the ARS definition is little sensitive to variation of these parameters.

*ARS definition*

-the y axis is defined as the line through the centre of the circumference fitted on the most proximal portion available of diaphysis and the centre of the circumference fitted on the most distal end of the diaphysis (fig. S2.d);

-the origin is the projection on the y-axis of the centroid of the fibulo-talar selected area (fig. S2.e);

- the x axis is obtained by the cross product between the y axis and the normal to the plane fitted on the fibula-talar articular surfaces, pointing anteriorly (fig. S2. f)

- the z axis is orthogonal to the x and y axes, pointing to the right (fig. S2.g).


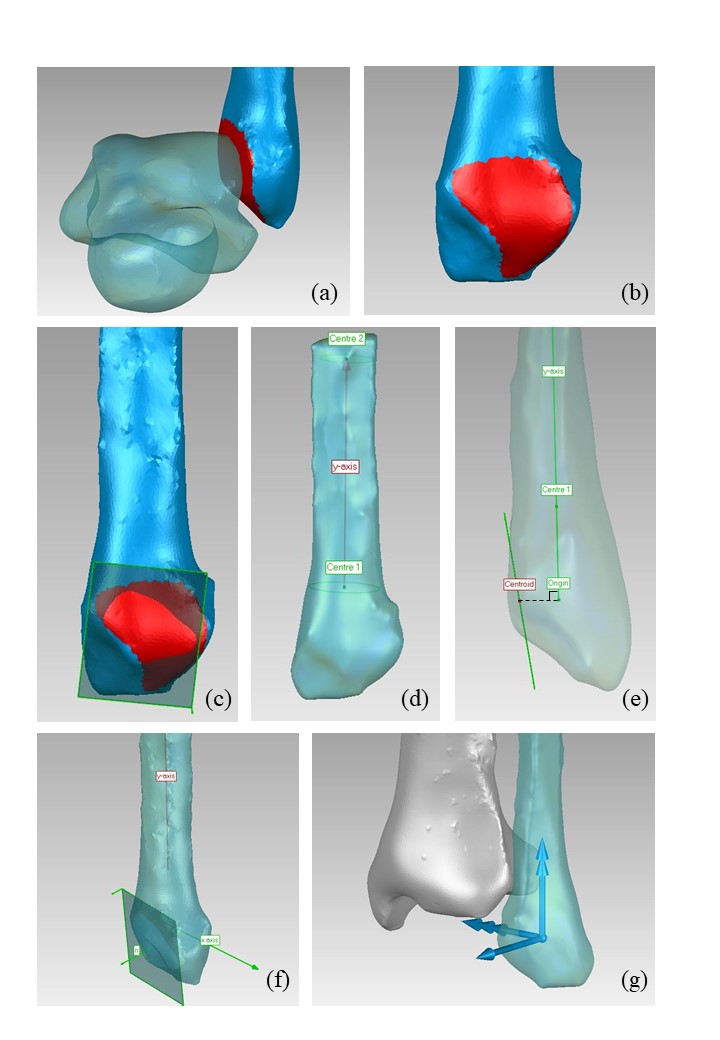


Fig. S2: Definition of the fibula ARS: a) identification of the fibulo-talar articular surface in coronal view; b) identification of the fibula-talar articular surface in sagittal view; c) plane fitted on the selected articular surface; d) identification of the y axis; e) identification of the origin; f) identification of the x axis; g) final ARS

Talus

| **Features to be identified** | **Parameters for the input file for the numerical code** |
| --- | --- |
| Cylinder fitted on the superior aspect of the trochlea tali, i.e. excluding the medial and lateral facies malleolaris | Centre of medial circumference |
|  | Centre of lateral circumference |

The identification of the cylinder fitted on the trochlea tali (fig. S3.a-d) is quite stable with respect to the area selection. Nevertheless, considering the importance of the talus, it is recommended to be consistent in the selection, taking particular care to the border of the selection.

*ARS definition*

- the z axis is coincident with the axis of the cylinder fitted on the trochlea tali, pointing to the right (fig. S3.d);

- the origin is at the midpoint of the height of the same cylinder (fig. S3.e);

- as for the y axis, the talus principal axes of inertia are computed; the axis forming the smallest angle with the tibia x axis is chosen and its cross product with the z axis is taken, pointing proximally.

- the x axis is orthogonal to the y and z axes, pointing anteriorly (fig. S3.f-g).


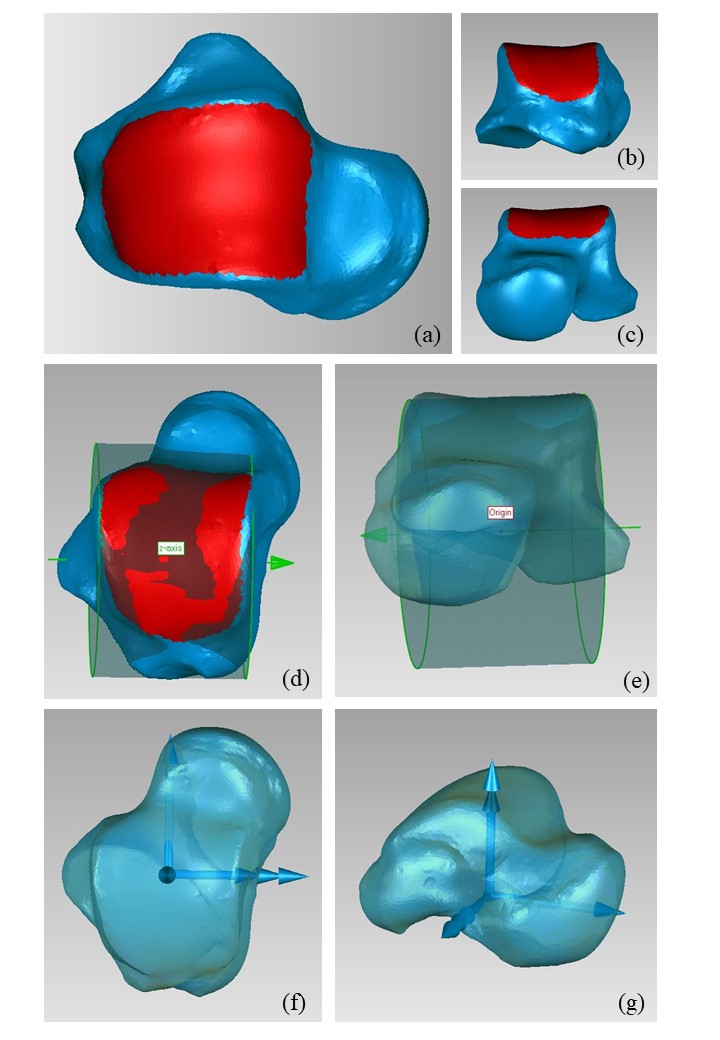


Fig. S3: Definition of the talus ARS: a-c) identification of the trochlea tali; d) cylinder fitted on trochlea tali; e) origin definition; f-g) final ARS.

Calcaneus

| **Features to be identified** | **Parameters for the input file for the numerical code** |
| --- | --- |
| Sphere fitted on both the anterior and middle talar facets | Centre of the sphere |
| Sphere fitted on the posterior talar facet | Centre of the sphere |
| Plane fitted on the surface articulating with the cuboid | Normal to the plane |

The identification of the sphere fitting the anterior and middle talar facets can be quite sensitive to outliers; in other terms, extending the selection beyond the actual articular surface may change considerably the location of the sphere centers. For this reason, it is recommended to be consistent in the selection, taking particular care to the border of the selection.

The normal to the plane fitted on the surface articulating with the cuboid must be oriented anteriorly or, in other words, toward the outside of the bone.

*ARS definition*

- the origin is coincident with the bone centroid;

- the x axis is parallel to the line through the centers of the spheres fitted on articular surfaces (see the feature description), pointing anteriorly (fig. S4.a-f);

- the z axis is defined as the cross product between the x axis and the normal to the plane fitting to the articulating surface between calcaneus and cuboid, pointing to the right (fig. S4.g-h);

- the y axis is orthogonal to the x and z axes, pointing proximally (fig. S4.i).


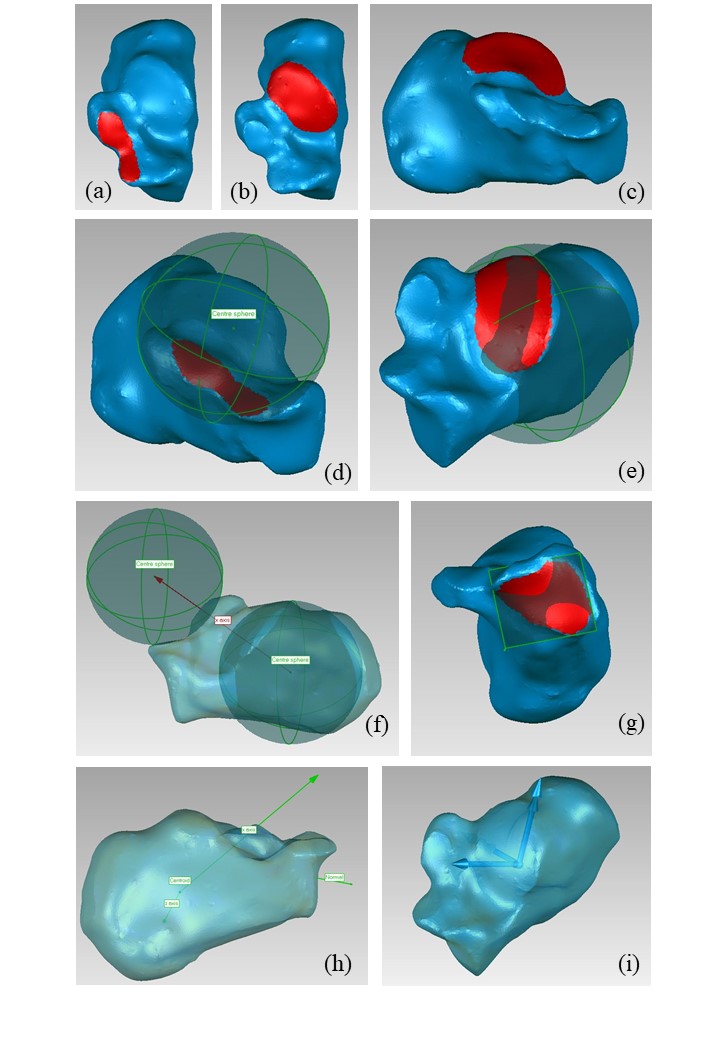


Fig. S4: Definition of the calcaneus ARS: a) identification of the sphere fitted on the anterior and middle talar facets; b-c) identification of the posterior talar facet; d) sphere fitted on anterior and middle talar facets; e) sphere fitted the on posterior talar facet; f) x axis definition; g) the plane fitted to the calcaneo-cuboidal articular surface; h) definition of the z axis; i) final ARS.

Navicular

| **Features to be identified** | **Parameters for the input file for the numerical code** |
| --- | --- |
| Sphere fitted on articular surface between navicular and talus | Centre of the sphere |

Identification of the talo-navicular articular surface is stable among operators and should present no specific challenges.

*ARS definition*

- the origin is coincident with the bone centroid;

- the x axis is defined as the axis through the origin and the centre of the sphere fitted on articular surface between navicular and talus, pointing anteriorly (fig. S5.a-c);

- as for the y axis, the navicular principal axes of inertia are computed; the axis forming the smallest angle with the talus z axis is chosen and its cross product with the x axis is taken, pointing proximally;

- the z axis is orthogonal to the x and y axes, pointing to the right (fig. S5.d),


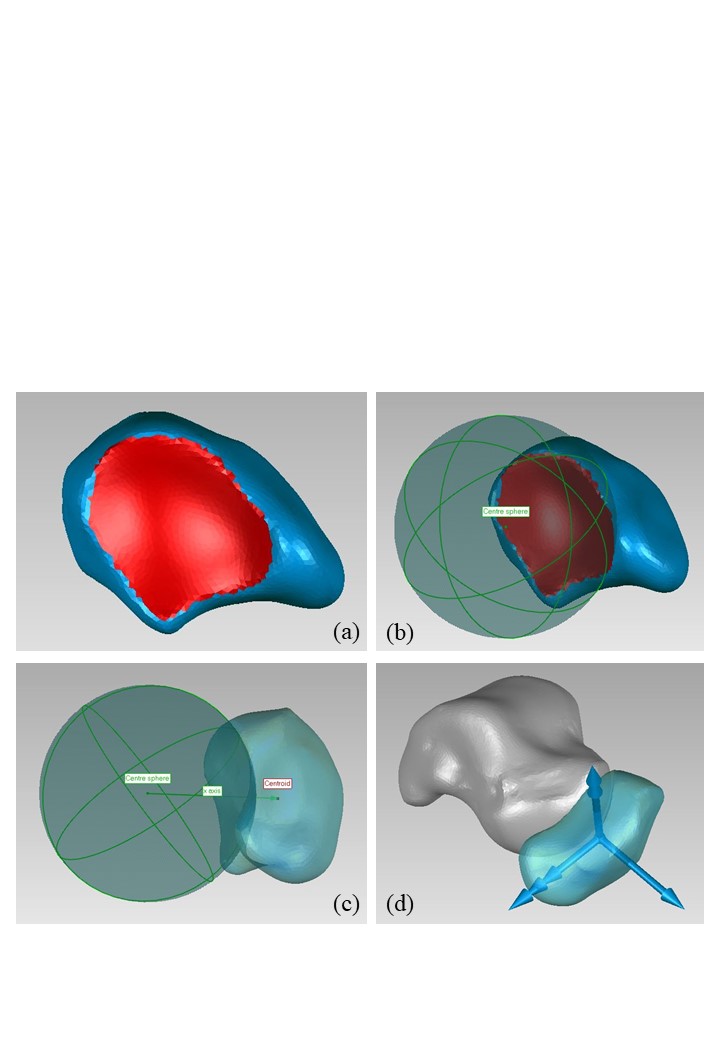


Figure S5: Definition of the navicular ARS: a) identification of the articular surface between navicular and talus; b) sphere fitted on articular surface between navicular and talus; c) x axis definition; d) final ARS.

Medial cuneiform

| **Features to be identified** | **Parameters for the input file for the numerical code** |
| --- | --- |
| Plane fitted on the anterior surface articulating with the first metatarsal | Normal to the plane |
| Plane fitted on the lateral surface articulating with the intermediate cuneiform and second metatarsal | Normal to the plane |

While the identification of the anterior surface articulating with the first metatarsal present small variability among the operators, the identification of the lateral surface articulating with intermediate cuneiform and second metatarsal can be less straightforward. However, the impact of this variability on the ARS definition is minor.

Both the normals to fitting planes must point toward the outside of the bone.

*ARS definition*

- the origin is coincident with the bone centroid.

- the x axis is the normal to the plane fitted on anterior surface articulating with the first metatarsal (fig. S6.a-b);

- the y axis is defined by the cross product between the normal to the plane fitted on lateral surface articulating with the intermediate cuneiform and the x axis, pointing proximally (fig. S6.c-e);

- the z axis is orthogonal to the x and y axes, pointing to the right (fig. S6.f).


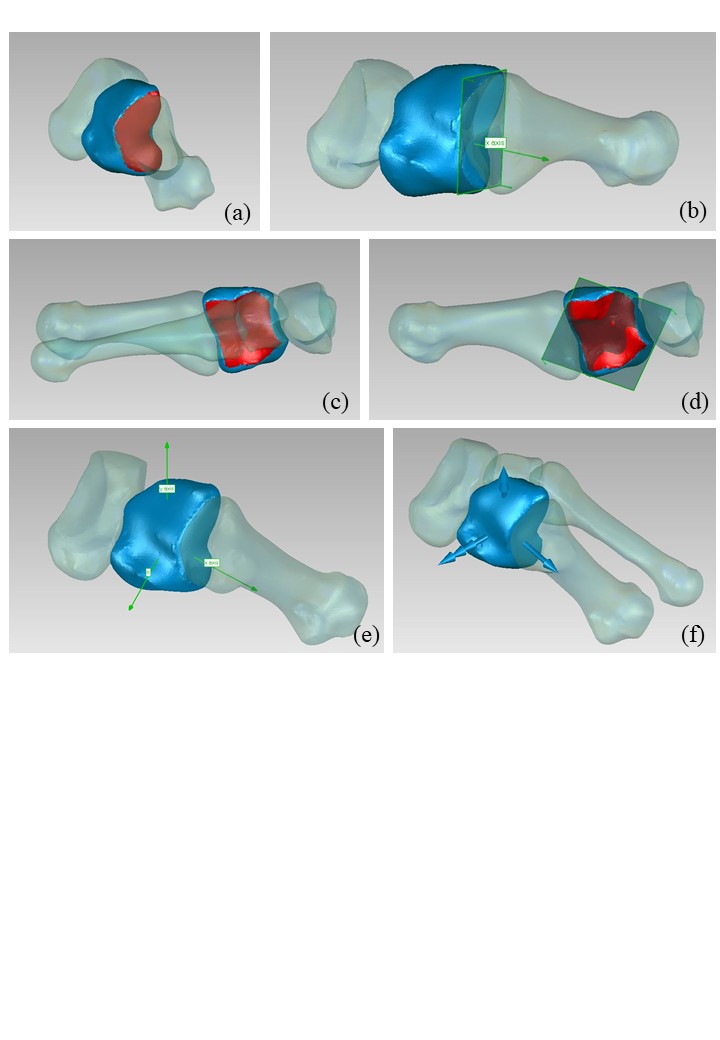


Figure S6: Definition of the medial cuneiform ARS: a) identification the surface articulating with first metatarsal; b) plane fitted on the same surface and identification of the x axis; c) identification of the surface articulating with the intermediate cuneiform and second metatarsal; d) plane fitted on the same surface; e) identification of the y axis; f) final ARS.

Intermediate cuneiform

| **Features to be identified** | **Parameters for the input file for the numerical code** |
| --- | --- |
| Plane fitted on the anterior surface articulating with the second metatarsal | Normal to the plane |
| Plane fitted on the posterior surface articulating with the navicular | Normal to the plane |
| Plane fitted on the medial surface articulating with the medial cuneiform | Normal to the plane |
| Plane fitted on the lateral surface articulating with the lateral cuneiform | Normal to the plane |

The identification of the articular surfaces for the plane fitting present no specific challenge for this bone. All the normals to fitting planes must point toward the outside of the bone.

*ARS definition*

- the origin is coincident with the bone centroid;

- the x axis is the mean between the normal to the plane fitted on the anterior surface articulating with the second metatarsal and the normal to the plane fitted on posterior surface articulating with the navicular (flipped in the code to compute mean direction) (fig. S7.a-c);

- as for the y axis, the mean between the normal to the plane fitted on the medial surface articulating with the medial cuneiform and the normal to the plane fitted on the lateral surface articulating with the lateral cuneiform is computed (both normal are taken pointing to the right in the code to compute mean direction); its cross product with the x axis is taken, pointing proximally (fig. S7.f);

- the z axis is orthogonal to the x and y axes, pointing to the right (fig. S7.g).


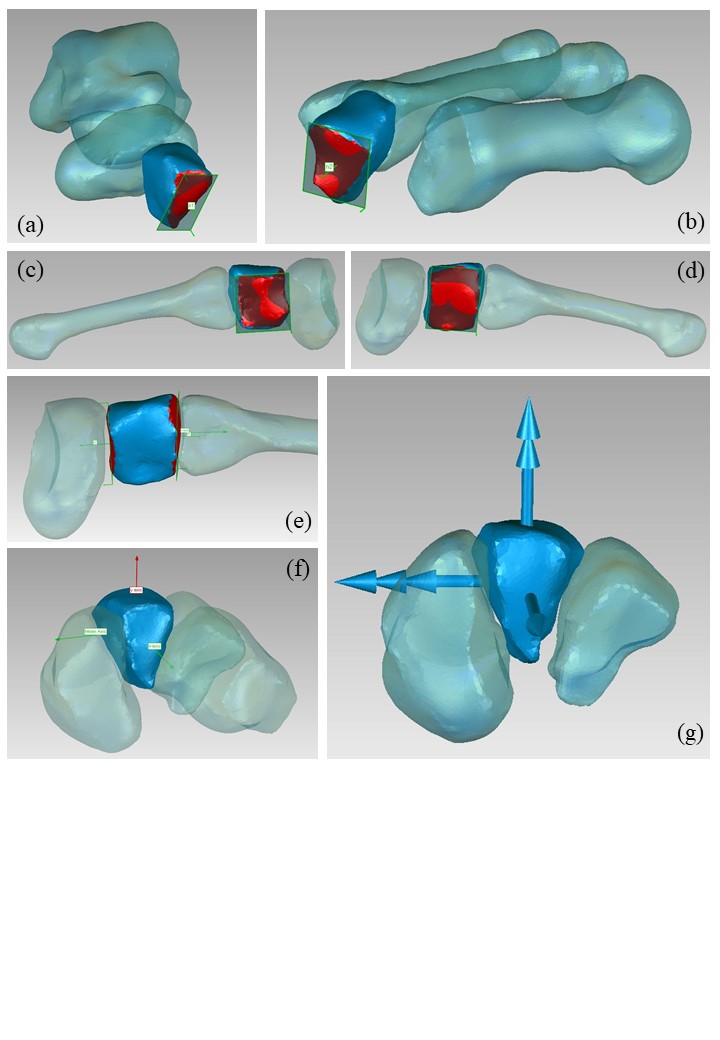


Figure S7: Definition of the intermediate cuneiform ARS: a) identification of the surface articulating with second metatarsal and fitted plane; b) identification of the surface articulating with navicular and fitted plane; c) identification of the surface articulating with the lateral cuneiform and fitted plane; d) identification of the surface articulating with the medial cuneiform and fitted plane; e) x axis definition; f) y axis definition; g) final ARS.

Lateral cuneiform

| **Features to be identified** | **Parameters for the input file for the numerical code** |
| --- | --- |
| Plane fitted on the anterior surface articulating with the third metatarsal | Normal to the plane |
| Plane fitted on the posterior surface articulating with the navicular | Normal to the plane |
| Plane fitted on the medial surface articulating with the lateral cuneiform and second metatarsal | Normal to the plane |
| Plane fitted on the lateral surface articulating with the cuboid | Normal to the plane |

The identification of the articular surfaces for the plane fitting presents no specific challenge for this bone. All the normals to fitting planes must point toward the outside of the bone.

*ARS definition*

- the origin is coincident with the bone centroid;

- the x axis is the mean between the normal to the plane fitted on the anterior surface articulating with the third metatarsal and the normal to the plane fitted on the posterior surface articulating with the navicular (flipped in the code to compute mean direction) (fig. S8.a-e);

- as for the y axis, the mean between the normal to the plane fitted on medial surface articulating with the intermediate cuneiform and the normal to the plane fitted on lateral surface articulating with the cuboid, both taken as pointing to the right, is computed (both normal are taken pointing to the right in the code to compute mean direction); its cross product with the x axis is taken, pointing proximally (fig. S7.f);

- the z axis is orthogonal to the x and y axes, pointing to the right (fig. S7.g).


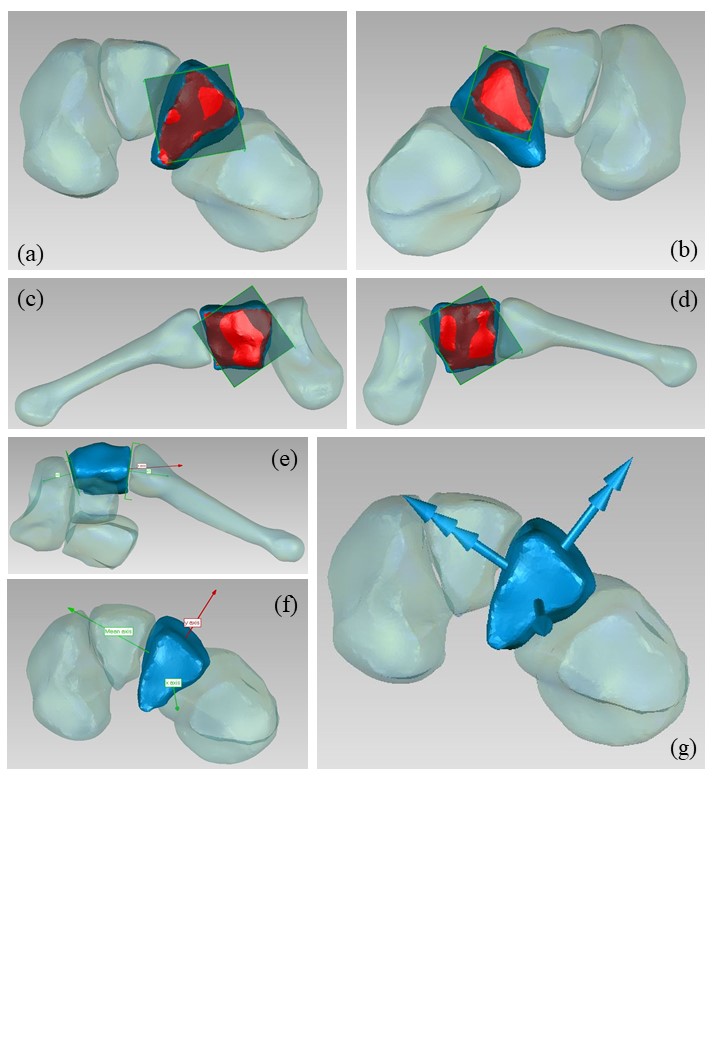


Figure S8: Definition of the lateral cuneiform ARS: a) identification of the surface articulating with the third metatarsal and fitted plane; b) identification of the surface articulating with the navicular and fitted plane; c) identification of the surface articulating with the cuboid and fitted plane; d) identification of the surface articulating with the intermediate cuneiform and fitted plane; e) x axis definition; f) y axis definition; g) final ARS.

Cuboid

| **Features to be identified** | **Parameters for the input file for the numerical code** |
| --- | --- |
| Plane fitted on the anterior surface articulating with the fourth and the fifth metatarsal | Normal to the plane |
| Plane fitted on the medial surface articulating with lateral cuneiform and navicular | Normal to the plane |

The identification of the articular surfaces for the plane fitting presents no specific challenge for this bone. All the normals to fitting planes must point toward the outside of the bone.

*ARS definition*

- the origin is coincident with the bone centroid.

- the x axis is the normal to the plane fitted on the anterior surface articulating with the fourth and fifth metatarsal (fig. S9.a-b);

- the y axis is given by the cross product between the normal to the plane fitted on medial surface articulating with the lateral cuneiform and navicular and the x axis, pointing proximally (fig. S9.c-d);

- the z axis is orthogonal to the x and y axes, pointing to the right (fig. S9.e).


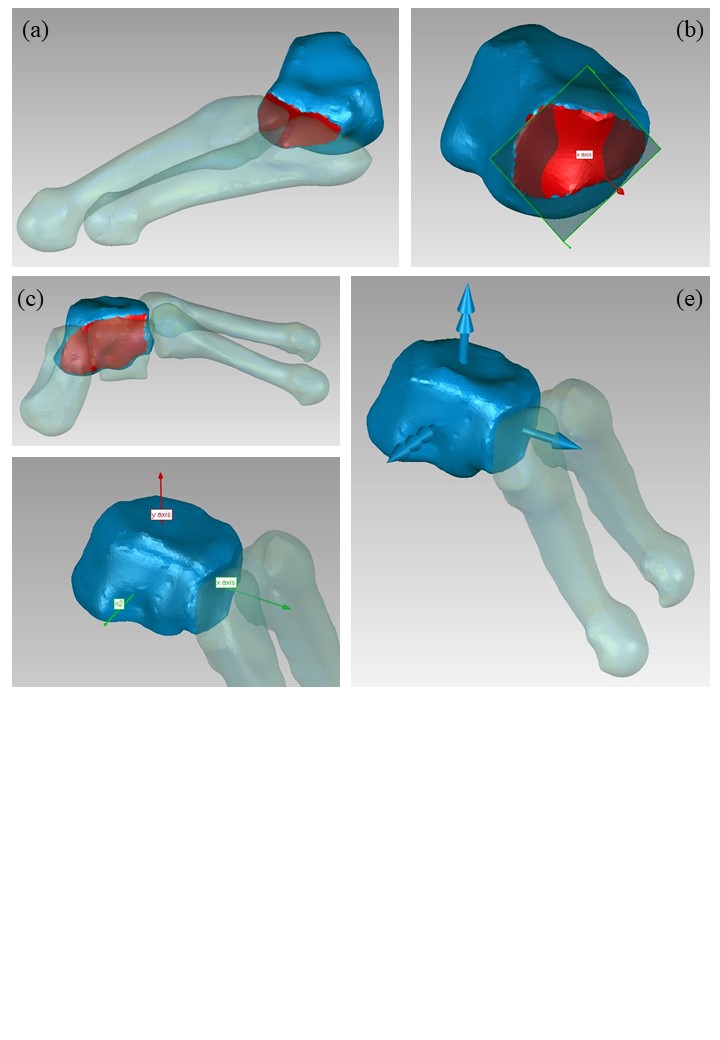


Figure S9: Definition of the cuboid ARS: a) identification of the surface articulating the fourth and the fifth metatarsal; b) plane fitted on the same surface and identification of the x axis; c) identification of the surface articulating with the navicular and fitted plane; c) identification of the surface articulating with the lateral cuneiform and navicular; d) plane fitted on the same surface and identification of the z axis; e) final ARS.

Metatarsal bones

Metatarsal ARS are automatically determined based on principal axes of inertia, sorted and oriented based on the proximal bone each metatarsal articulates with. In what follows the definitions for each metatarsal are reported.

*ARS definition: first metatarsal (fig. S10.a)*

- the origin O is the centroid of the bone;

- the x axis is the bone first principal axis of inertia, pointing anteriorly, orientation is determined to minimize the angle with the medial cuneiform x axis;

- the y axis is the bone principal axis of inertia showing the minimum angle with the medial cuneiform y axis, pointing proximally;

- the z axis is orthogonal to the x and y axes, pointing to the right.

*ARS definition: second metatarsal (fig. S10.b)*

- the origin O is the centroid of the bone;

- the x axis is the bone first principal axis of inertia, pointing anteriorly, orientation is determined to minimize the angle with the intermediate cuneiform x axis;

- the y axis is the bone principal axis of inertia showing the minimum angle with the intermediate cuneiform y axis, pointing proximally;

- the z axis is orthogonal to the x and y axes, pointing to the right.

*ARS definition: third metatarsal (fig. S10.c)*

- the origin O is the centroid of the bone;

- the x axis is the bone first principal axis of inertia, pointing anteriorly, orientation is determined to minimize the angle with the lateral cuneiform x axis;

- the y axis is the bone principal axis of inertia showing the minimum angle with the lateral cuneiform y axis, pointing proximally;

- the z axis is orthogonal to the x and y axes, pointing to the right.

*ARS definition: fourth and fifth metatarsal (fig. S10.d-e)*

- the origin O is the centroid of the bone;

- the x axis is the bone first principal axis of inertia, pointing anteriorly, orientation is determined to minimize the angle with the cuboid x axis;

- the y axis is the bone principal axis of inertia showing the minimum angle with the cuboid y axis, pointing proximally;

- the z axis is orthogonal to the x and y axes, pointing to the right.


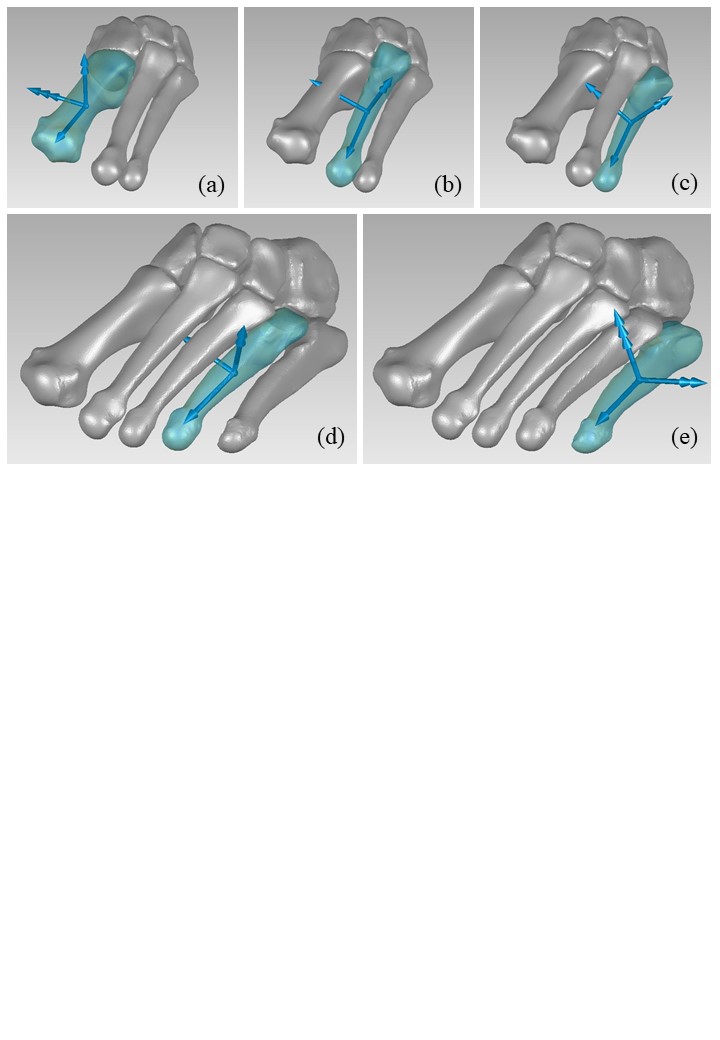


Figure S10: First (a), second (b), third (c), fourth (d), and fifth (c) metatarsal ARS.
